# Role of ABCC5 in cancer drug resistance and its potential as a therapeutic target

**DOI:** 10.3389/fcell.2024.1446418

**Published:** 2024-11-05

**Authors:** Yinlong Pan, Mengmeng Wu, Huazhong Cai

**Affiliations:** ^1^ Department of Emergency, Affiliated Hospital of Jiangsu University, Zhenjiang, China; ^2^ Department of Anesthesiology, Affiliated Hospital of Jiangsu University, Zhenjiang, China

**Keywords:** ABCC5, cancer, drug resistance, therapeutic target, transporters

## Abstract

Over 90% of treatment failures in cancer therapy can be attributed to multidrug resistance (MDR), which can develop intracellularly or through various routes. Numerous pathways contribute to treatment resistance in cancer, but one of the most significant pathways is intracellular drug efflux and reduced drug concentrations within cells, which are controlled by overexpressed drug efflux pumps. As a member of the family of ABC transporter proteins, ABCC5 (ATP Binding Cassette Subfamily C Member 5) reduces the intracellular concentration of a drug and its subsequent effectiveness using an ATP-dependent method to pump the drug out of the cell. Numerous studies have demonstrated that ABCC5 is strongly linked to both poor prognosis and poor treatment response. In addition, elevated ABCC5 expression is noted in a wide variety of malignancies. Given that ABCC5 is regulated by several pathways in a broad range of cancer types, it is a prospective target for cancer treatment. This review examined the expression, structure, function, and role of ABCC5 in various cancer types.

## Introduction

As the second leading cause of global mortality, cancer accounted for 600,000 deaths in the United States in 2018 (Narayanan et al., 2020). Multidrug resistance (MDR) to chemotherapy, which is characterized by resistance to numerous medicines with diverse structures and modes of action, is a significant obstacle in cancer treatment. MDR in tumour cells, whether endogenous or acquired, significantly reduces the therapeutic efficacy of medications. MDR is linked to antiapoptotic processes, survival, genetic mutations, absorption defects, and excessive drug export from cells (Hoffmann and Lambert, 2014). ABC transporter proteins are powered by ATP hydrolysis to pump certain chemicals and drugs out of the cell, thus promoting the formation of MDR through excessive export of intracellular antitumor drugs (Deeley et al., 2006; Wilkens, 2015).

Because members of the ABCC subfamily of the ABC family promote resistance in several malignancies, they are occasionally referred to as multidrug resistance proteins (MRPs) (Deeley et al., 2006). The ABCC subfamily contains nine members (ABCC1-6 and ABCC10-12, respectively). Most tissues exhibit widespread expression of ABCC5/MRP5, with the brain and muscle exhibiting very high levels (Belinsky et al., 1998; Kool et al., 1997; McAleer et al., 1999). ABCC5 is a generalized glutamate-coupled and analogue transporter and acts as an organic anion pump that can transport nucleoside analogues (Jansen et al., 2015a; Wijnholds et al., 2000). ABCC5 mediates drug resistance and promotes tumour proliferation and metastasis in different types of cancer (Chen et al., 2022; Chen et al., 2021a). Fewer studies have been conducted on ABCC5, and there is minimal literature detailing the role of ABCC5 in cancer drug resistance. Current research on the ABC family has focused on breast cancer resistance protein (BCRP), multidrug resistance-associated protein 1 (MRP1), and p-glycoprotein (P-gp). The primary aim of this review is to address this gap by providing a comprehensive overview of the current understanding of ABCC5’s structure, function, and its role in mediating drug resistance across various malignancies. We will specifically focus on its involvement in cancers such as breast, lung, liver, colorectal, pancreatic, ovarian, prostate, cervical, and nasopharyngeal cancers. Additionally, we will discuss ABCC5’s potential as a therapeutic target for overcoming drug resistance in cancer treatment. By exploring the existing research and identifying areas that warrant further investigation, this review aims to contribute to the development of more effective strategies for combating cancer drug resistance.

## Drug resistance

The two main types of resistance to cancer therapy are acquired resistance (secondary) and intrinsic resistance (primary) (Marine et al., 2020). The characteristic of primary resistance is the absence of an objective clinical response to therapy. Conversely, following clinical remission, secondary resistance frequently occurs due to distant or localized recurrence of malignant tumours (Marine et al., 2020).

When considering drug delivery, one of the most important factors is to achieve maximum intracellular bioavailability of the drug for successful therapeutic effects over a longer period. Nevertheless, increasing data indicate that MDR acquired intracellularly or through multiple mechanisms may be a major barrier, accounting for more than 90% of treatment failures (Kankala et al., 2017). Many malignant tumours respond more strongly to chemotherapeutic agents during the early stages of treatment. However, as tumour cells develop MDR, the anticancer effect of chemotherapeutic agents is greatly reduced, and MDR has emerged as one of the major challenges in treating cancerous tumours. The achievement of MDR in cells involves several important mechanisms. These mechanisms include reduced drug uptake, active efflux, DNA repair, suppression of apoptosis, and modification of chemotherapeutic targets to prevent cell death (Kunjachan et al., 2013; Cong et al., 2020). The correlation between the emergence of MDR and reduced medication absorption, particularly for chemotherapeutic medicines, is becoming increasingly well acknowledged. This increase in drug efflux has been used by cells to increase their survival (Bukowski et al., 2020). Multiple uptake mechanisms, including diffusion and passive and active transport pathways, are generally advantageous for medication absorption. Drug efflux pumps on the cell surface are overexpressed, resulting in the adaptation of internalized efflux of a variety of chemotherapeutic medicines (Duan et al., 2023). This is the major mechanism leading to MDR. ABCC5 is derived from the ABCC subfamily which contains 9 members (ABCC1-6 and ABCC10-12 respectively). Most cancer cells express ABCC5. BCPR, P-gp, and MRP1 are the efflux pumps and are members of the ABC family of transporter proteins (Davis and Sherbenou, 2021). P-gp, the most researched ABC transporter protein in tumour MDR, controls intracellular drug efflux to prevent cells from dying from excessive intracellular drug concentrations, hence mediating the emergence of drug resistance. The intracellular concentrations of numerous chemotherapeutic medications, including vinblastine alkaloids (vinblastine), taxanes (paclitaxel), and anthracycline medications (daunorubicin), are reduced when P-gp is overexpressed. In addition, drug resistance has been linked to genetic changes in ABC family transporter proteins (Catalano et al., 2022). Many recent investigations have provided evidence of the role of ABCC5 in drug resistance in different forms of cancer (Zhu et al., 2023; Wojtowicz and Nowicki, 2023; Strachowska et al., 2023; El-Daly et al., 2023).

## Structure, function, expression, and substrates of ABCC5

### Structure

**FIGURE 1:**
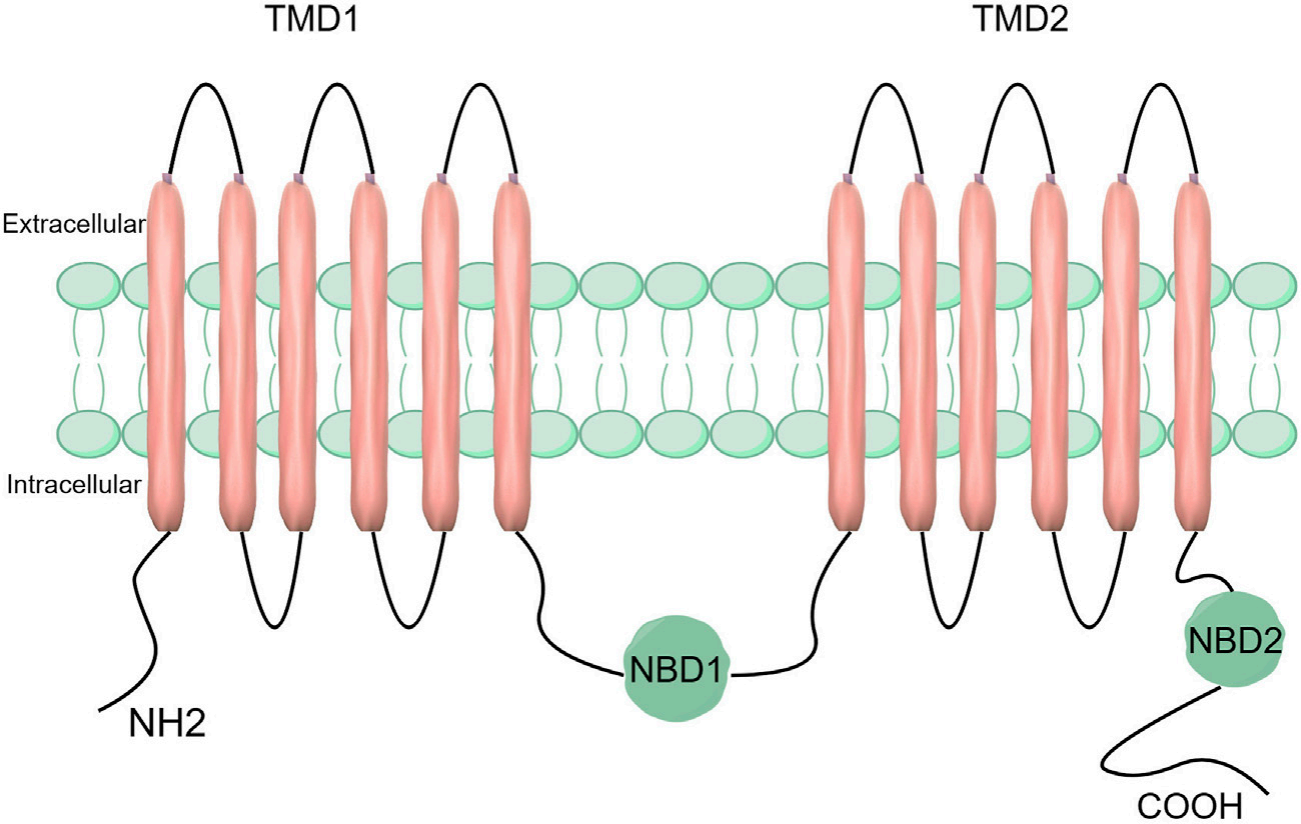
Structure of the ABCC5 transporter protein. ABCC5 consists of two TMDs and two NBDs.

ABCC5 is composed of two hydrophobic transmembrane structural domains (TMD 1 and TMD 2) and two highly conserved hydrophilic nucleotide-binding structural domains (NBD1 and NBD2) (Choudhuri and Klaassen, 2006). Whereas the TMD is responsible for substrate binding and translocation, the NBD is responsible for ATP hydrolysis and binding. The NBD functions as a source of energy to propel substrate transport and is composed of both ATP-binding and ATP-hydrolysing sites (Hohl et al., 2012). The function of TMD is to transport and bind substrates. It has transmembrane channels and substrate binding sites and uses a transmembrane α-helix structure to transport substrates from intracellular to extracellular spaces (Figure 1) (Choudhuri and Klaassen, 2006; Beis, 2015). The Walker A and Walker B motifs and the ABC signature motifs are among the significant structural features found in the highly conserved NBD structural domain (Hohl et al., 2012). The NBD structural domain is a dimer formed by the binding of two complementary NBD monomers using ATP. The γ-phosphate moiety of ATP is located between one NBD’s ABC signature motif and the other NBD’s Walker A motif (Hohl et al., 2012). The catalytic site of the protein is formed by the ABC characteristic motif, a unique sequence found in the alpha helix structural domain (Wilkens, 2015; Hohl et al., 2012).

### Function

The biological function of ABCC5 has not been fully elucidated, and current evidence suggests that ABCC5 functions in regulating intracellular cyclic nucleotide levels under some circumstances, such as decreased phosphodiesterase activity or guanylate cyclase inhibition (Wielinga et al., 2003). Hyaluronic acid transport to the extracellular matrix might involve ABCC5, and the process may be controlled by cGMP levels (Schulz et al., 2007). Studies have shown that ABCC5 can reduce the anticancer efficacy of doxorubicin, 6-thioguanine (6-TG), 6-mercaptopurine (6-MP), paclitaxel, 5-FU, celecoxib, irinotecan and 9-(2-phosphonomethoxyethyl) adenine (PMEA) (Wang et al., 2021a). It has been reported that ABCC5 contributes to bone metastasis in breast cancer by increasing the generation of osteoclasts and encouraging osteolytic bone breakdown (Mourskaia et al., 2012). In addition, ABCC5 is a signalling protein that mediates physiological functions (Russel et al., 2008). However, the physiological function of ABCC5 is not well understood. Experiments have shown that mice with ABCC5 allele knockout are still healthy and fertile, but this finding does not necessarily indicate that ABCC5 has no physiological function (Wijnholds et al., 2000).

### Expression

The majority of human organs contain ABCC5, with the heart, brain, and skeletal muscle showing especially high concentrations of the protein (Selim et al., 2023). Analysis of the human placental expression pattern of ABCC5 at the protein and mRNA levels revealed that the mid-gestation placenta (23–32 weeks gestation in the early stage and 32–37 weeks gestation in the late preterm stage) had higher ABCC5 mRNA levels than did the full term placenta (Meyer Zu Schwabedissen et al., 2005). Zhang et al. demonstrated that ABCC5 is abundantly expressed in both human and rodent cerebral arteries and plays a role in the barrier function of drug transport in the brain. It appears that the majority of cells express ABCC5 in their basolateral plasma membranes, whereas blood‒brain barrier cells express ABCC5 in their parietal membranes (Jansen et al., 2015a). ABCC5 is primarily found in the vascular smooth muscle cells of the heart, myocardial cells, and vascular endothelial cells. Compared to the ventricular samples, the auriculoventricular samples had lower ABCC5 mRNA levels. Under ischaemic conditions, ABCC5 expression in the ventricle was upregulated, which could be connected to ischaemic preconditioning. cGMP levels are increased in ischaemic tissue to enhance cGMP elimination, whereas ABCC5 enhances cGMP efflux in cells (Dazert et al., 2003).

Furthermore, numerous investigations have demonstrated that abnormally elevated ABCC5 expression is present in a multitude of malignant cancers. A study used breast cancer cell lines with different metastatic potentials, including human MDA-MB-231 (bone and lung metastatic sublines) and mouse 4T1 (non-metastatic and lung metastatic sublines), to show that ABCC5 expression is highest in bone metastatic cells (Mourskaia et al., 2012). Cells were cultured in high-glucose DMEM with 10% FBS. ABCC5 was knocked down using lentivirus-mediated shRNA, and cells were injected into mice for monitoring via bioluminescence imaging and micro-CT to assess bone metastasis and destruction (Mourskaia et al., 2012). ABCC5 expression was confirmed by Western blotting and RT-qPCR (Mourskaia et al., 2012). The findings indicate that ABCC5 was most highly expressed in breast cancer cells exhibiting a bone metastasis phenotype in the absence of therapy (anti-oestrogen, chemotherapy, or bisphosphonates) (Mourskaia et al., 2012). Breast cancer cells that develop bone metastases overexpress ABCC5 (Mourskaia et al., 2012). In contrast, ABCC5 is poorly expressed in hepatocytes (Wang et al., 2021b). ABCC5 expression is upregulated by toxic allyl alcohol therapy or total parenteral nutrition (Meyer Zu Schwabedissen et al., 2005). Hepatocellular carcinoma displays increased ABCC5 expression, and high ABCC5 expression levels are linked to a poor prognosis in hepatocellular carcinoma patients according to single-cell and pancancer analyses (Chen et al., 2021a). ABCC5 expression is notably increased in prostate cancer compared with benign prostatic hyperplasia (Chen et al., 2022).

### Substrate

Currently, ABCC5 has fewer known substrates, most of which are endogenous metabolites, including folic acid, cyclic nucleotides, and N-lactoyl amino acids (Wielinga et al., 2005; Jedlitschky et al., 2000; Jansen et al., 2015b). In addition, ABCC5 also transports purine derivatives, including PMEA and 6-mercaptopurine (Zhou et al., 2008). Drug resistance is associated with the ability of ABCC5 to transport antifolate and nucleotide analogues (Wielinga et al., 2005; Reid et al., 2003). A study has shown that ABCC5 is a universal analogue transporter and glutamate conjugate, and the chemical structures of known ABCC5 substrates (folic acid, methotrexate, and its polyglutamine metabolites) have been assessed. In addition to glutamic acid and aspartic acid, other compounds with glutamic acid or aspartic acid moieties (this study tested only N-acetyl glutamic acid; the excitatory toxic glutamate analogues kainic acid, NMDA and domoic acid; the therapeutic glutamate analogue ZJ43; and the tetrapeptide Val-Asp-Gly-Glu) are also substrates of ABCC5 (Jansen et al., 2015a). We summarized the substrates of ABCC5 and organized them into Table 1.

**TABLE 1:** The substrates of ABCC5.

Substrate	Type	Ref
Folic acid	Endogenous metabolites	Wielinga et al. (2005), Jedlitschky et al. (2000), Jansen et al. (2015b)
cGMP	Endogenous metabolites	Wielinga et al. (2005), Jedlitschky et al. (2000), Jansen et al. (2015b)
cAMP	Endogenous metabolites	Wielinga et al. (2005), Jedlitschky et al. (2000), Jansen et al. (2015b)
6-Mercaptopurine	Purine derivatives	Wijnholds et al. (2000)
6-Thioguanine	Purine derivatives	Wijnholds et al. (2000)
PMEA	Purine derivatives	Wijnholds et al. (2000)
Cladribine	Nucleotide analogues	Zhou et al. (2008)
Abacavir	Nucleoside analog	Zhou et al. (2008)
Heme	Endogenous metabolites	Korolnek et al. (2014)
N-lactoyl-amino acids	Endogenous metabolites	Jansen et al. (2015b)
NMDA	Excitotoxic glutamate analog	Jansen et al. (2015a)
Kainic acid	Excitotoxic glutamate analog	Jansen et al. (2015a)
Domoic acid	Excitotoxic glutamate analog	Jansen et al. (2015a)
ZJ43	Therapeutic glutamate analog	Jansen et al. (2015a)
Val-Asp-Gly-Glu	Tetrapeptide	Jansen et al. (2015a)

## ABCC5 and cancer and its resistance to treatment

In cancer cells, the overexpression of drug efflux pumps reduces drug concentrations and significantly reduces the therapeutic benefits of chemotherapy treatments (Zhang et al., 2023b). The overexpression of multiple drug efflux pumps contributes to the development of resistance in many cancer types (Duan et al., 2023). According to numerous studies, ABCC5 is expressed extensively in tumour tissues and is associated with both drug resistance and prognosis in many cancer types (Zhang et al., 2021). Next, we will discuss the relationship among ABCC5, cancer and drug resistance in a variety of cancers.

### Breast cancer

**FIGURE 2:**
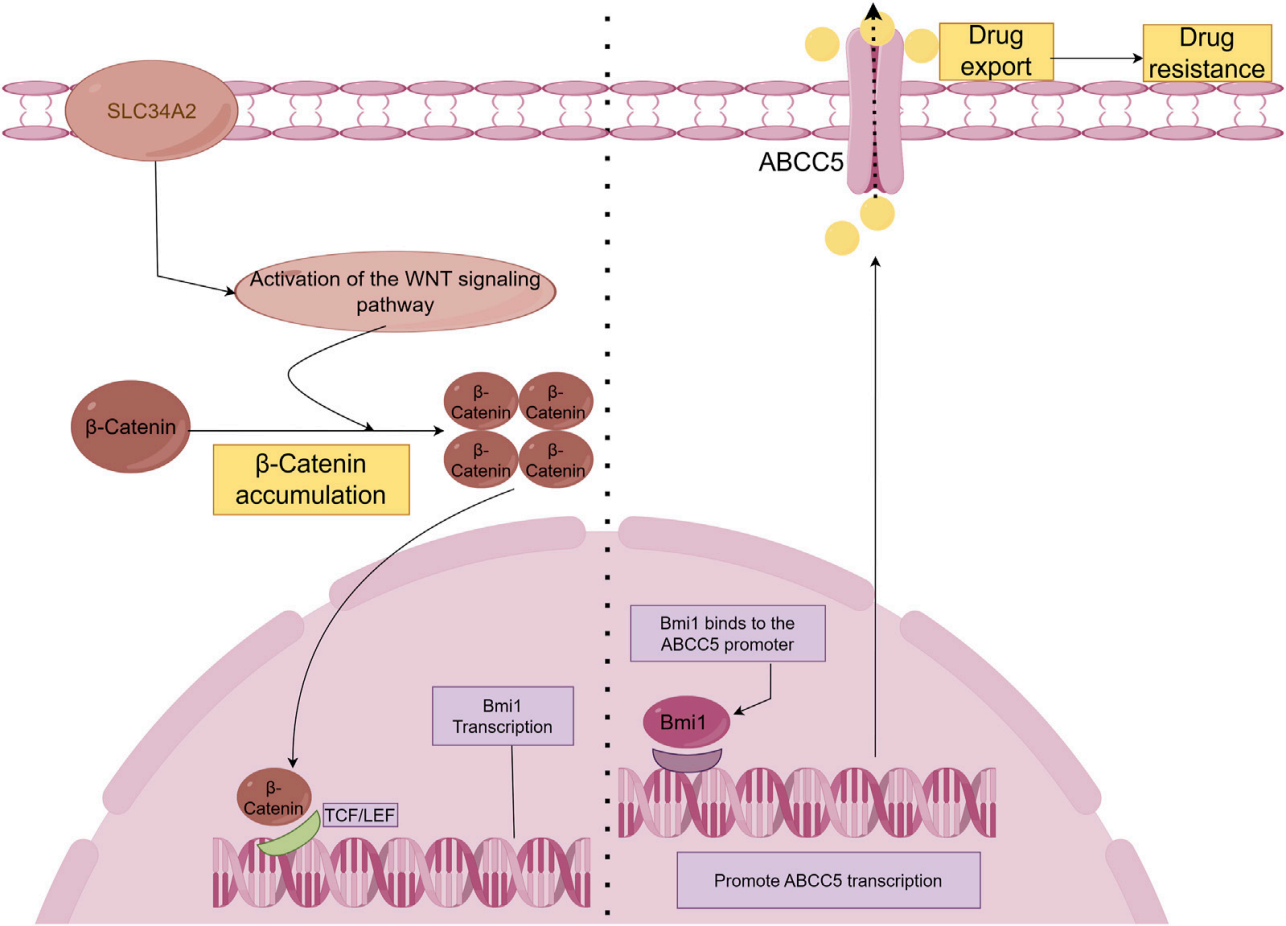
SLC34A2-Bmi1-ABCC5 signalling pathway hypothesis diagram. Studies have shown that SLC34A2 is located upstream of Bmi1, and ABCC5 is a direct transcription target of Bmi1. Bmi1 directly binds to and activates the human ABCC5 promoter and promotes ABCC5 transcription (Ge et al., 2016). Overexpression of ABCC5 promotes drug efflux and induces chemical drug resistance. However, this study has not yet explained the mechanism by which SLC34A2 regulates Bmi1. Some articles have pointed out that SLC34A2 regulates Bmi1 through the Wnt/β-Catenin pathway to promote the tumorigenic ability and self-renewal ability of LCSCs (Jiang et al., 2016). Therefore, we hypothesized that β-Catenin accumulates in the cell and enters the nucleus after SLC34A2 activates the Wnt pathway, and binds to T-cell factor/lymphoid enhancer factor (TCF/LEF) to activate Bmi1 transcription under the combined action of multiple proteins.

The SLC34A2-Bmi1-ABCC5 signalling pathway is responsible for inducing chemoresistance in breast cancer stem cell-like cells (BCSCs). The type II Na/Pi cotransporter protein NaPi2b is encoded by the SLC34A2 gene, which belongs to the solute carrier gene family (Xu et al., 1999). Bmi-1 is a stem cell-associated transcription factor and is associated with chemotherapy resistance (Awasthi et al., 2002; Awasthi et al., 2003). Researchers first obtained single-cell suspensions and isolated CD44^+^CD24^−^ cell populations using culture and screening methods. Next, the doxorubicin sensitivity of both non-CD44^+^ CD24^−^ cells and CD44^+^ CD24^−^ cells was assessed. Subsequently, researchers assessed SLC34A2, Bmi1, and ABCC5 gene expression levels. In addition, SLC34A2 cells were transduced with either siRNA or lentivirus to assess its effects on BCSCs. Finally, the effects of SLC34A2 on tumour growth and apoptosis were evaluated. The results revealed that SLC34A2 induces chemoresistance in BCSCs by activating Bmi1, which then increases ABCC5 expression. ABCC5 helps the cancer cells resist chemotherapy by pumping out drugs, making the cells less responsive to treatments like doxorubicin (Ge et al., 2016). In addition, it has been shown that SLC34A2 regulates Bmi1 expression through Wnt/β-catenin pathway in lung cancer cells (Jiang et al., 2016). Therefore, we speculate that SLC34A2 in the SLC34A2-Bmi1-ABCC5 signalling pathway also regulates Bmi1 expression through the Wnt/β-catenin pathway (Figure 2).

ABCC5 is regulated by miR-128 to affect drug resistance of breast cancer. The expression of miR-128, which is a tumour suppressor gene, was originally found to be reduced in glioblastoma (Zhang et al., 2009). MiR-128 expression is abnormal in cancer cells, resulting in their malignant phenotype (Zhang et al., 2009). A study showed that reduced levels of miR-128 in breast tumour-initiating cells (BT-ICs) confer resistance to doxorubicin by reducing translational repression through targeting the 3′-UTR and increasing Bmi-1 and ABCC5 protein expression (Zhu et al., 2023). Through a series of experiments and analyses, it was found that breast cancer patients with decreased miR-128 expression had poorer prognoses and were more likely to develop chemotherapy resistance (Zhu et al., 2023). Additional mechanistic research revealed that the Bmi-1 and ABCC5 genes are regulated by miR-128, which impacts the chemosensitivity of breast cancer stem cells (Zhu et al., 2023). These findings provide new indicators of the treatment response and prognostic assessment of breast cancer and provide a basis for developing miR-128 mimics combined with chemotherapy treatment strategies.

The expression of ABCC5 could be inhibited by miR-21. For breast cancer patients, high miRNA-21 expression levels are associated with poor prognosis (Fang et al., 2017). According to a study that elucidated its mechanism of action, miR-21 causes breast cancer cells to proliferate and metastasize (Rama et al., 2022). In cell tests and animal models, researchers have shown that miR-21 expression is directly associated with breast cancer occurrence and progression (Rama et al., 2022). Bioinformatics analysis and experimental validation showed that miR-21 directly targeted ABCC5, and when miR-21 was overexpressed, breast cancer cells were more likely to regain sensitivity to doxorubicin by inhibiting the expression of ABCC5 (Rama et al., 2022). Additional research findings demonstrated that breast cancer patients with high miR-21 expression had a poorer prognosis (Rama et al., 2022).

Increased levels of GDF15 were detected in breast cancer tissues, as demonstrated by Modi et al. The positive correlation between GDF15 mRNA and ABCC5, and FOXM1 expression suggested that GDF15 is linked to drug resistance. Furthermore, triple-negative breast cancer tissues exhibit 9.1-fold increased ABCC5 expression and 5.13-fold increased GDF15 expression compared with breast cancer tissues (Modi et al., 2022).

In MCF7 cells, NRF2 coexpression with active HER2 (HER2CA) promoted the expression of the NRF2 target genes ABCC5. HER2 is an epidermal growth factor receptor (EGFR)/HER2 family receptor tyrosine kinase (RTK) (Moasser, 2007). One of the main transcriptional regulators, nuclear factor erythroid 2-related factor 2 (NRF2), activates genes associated with drug resistance, detoxification, and oxidative stress (Bryan et al., 2013; Niture et al., 2014). A group of genes, including ABCC5, that exhibit increased transcription when HER2CA binds to NRF2, are implicated in drug resistance in cancer cells (Kang et al., 2014). HER2 and NRF2 were overexpressed in breast cancer cells, treated with drugs, and then subject to cell proliferation experiments to assess the sensitivity of HER2 and NRF2 to drugs (Kang et al., 2014). Next, immunoprecipitation assays and DNA binding assays were used to determine the interaction between HER2 and NRF2 as well as the DNA binding capacity of NRF2 (Kang et al., 2014). In addition, the researchers analysed the transcriptional regulation of multiple genes by HER2 and NRF2 (Kang et al., 2014). Through some experiments, the researchers discovered that HER2 directly interacts with NRF2, increasing NRF2 stability and activity. Furthermore, HER2 and NRF2 coexpression led to the transcriptional activation of other drug resistance genes, such as ABCC5 (Kang et al., 2014).

ABCC5 gene overexpression caused breast cancer cells to become less sensitive to pemetrexed (MTA), accumulate less MTA, and exhibit increased efflux, preventing apoptosis. MTA, a novel multitargeted antifolate drug, is mainly used to treat locally advanced breast cancer (O'Shaughnessy et al., 2005). The positive correlation between MTA resistance in breast cancer and ABCC5 overexpression was validated using an *in vivo* xenograft model (Chen et al., 2021b). According to experimental findings from another study, liposomal MTA treatment has a greater antitumour impact than free MTA treatment in the MCF-7+ABCC5 xenograft model (Bai et al., 2018). This suggests that the development of liposomal MTA may be one of the strategies to inhibit ABCC5-mediated MTA resistance in breast cancer.

Statistical analysis of publicly available breast cancer datasets suggested that ABCC5 is a potential predictor of recurrent and metastatic breast cancer and may serve as a candidate gene for further study of breast carcinogenesis, invasion, metastasis, and epithelial–mesenchymal transition (Tapak et al., 2023). Furthermore, when overexpressed in breast cancer, ABCC5 can confer resistance to 5′-fluorouracil (5-FU) to tumour cells by transporting monophosphate metabolites (Pratt et al., 2005).

### Lung cancer

BRM270 may mediate the drug resistance of lung cancer through ABCC5. BRM270 is a formulated extract of seven plants that restrains the proliferation of a wide variety of cancer cells (Mongre et al., 2015). Upregulation of microRNA (miRNA)-128 expression in the A549 cell line regulates CSC self-renewal and tumour initiation. This finding suggested that the combination of miRNA-128 and BRM270 may be an effective treatment for chemotherapy-resistant non-small cell lung cancer (NSCLC) cells (Kwon et al., 2018). Researchers first treated NSCLC cell lines with BRM270 to assess its effects on cell proliferation and the expression of ALDH, a marker of tumour stem cells (Gu et al., 2021). After employing immunohistochemistry to identify ALDH1A1 expression in tumour tissues, statistical analyses were performed to compare the variations across groups. In addition to inhibiting NSCLC cell proliferation and increasing the number of lung cancer stem cells, BRM270 controls the transcriptional activity of miR-128 and downstream target genes (Gu et al., 2021). c-MET, ABCC5 and Bmi1 expression is inhibited by BRM270 (Gu et al., 2021). Another study demonstrated that BMI1 regulates ABCC5 transcription directly (Zhu et al., 2023). Therefore, we propose that the chemoresistance of NSCLC is mediated by BRM270, which decreases Bmi1 and ABCC-5 expression and increases miR-128 expression in the presence of endothelial repressors. However, this has not been demonstrated experimentally.

Trichostatin A (TSA) and suberoylanilide hydroxamic acid (SAHA), two histone deacetylase (HDAC) inhibitors, have been identified as new types of anticancer drugs given their potent anticancer properties (Xu et al., 2018; Jiang et al., 2013; Ni et al., 2015). In A549 cells, TSA and SAHA increase ABCC5 expression (Wang et al., 2019). The expression of ABC transporter proteins was determined in A549 and HCT116 cells using RT‒qPCR. The results demonstrated that TSA and SAHA significantly increased ABCC5 mRNA expression (Wang et al., 2019).

NSCLCs harbouring EGFR-activating mutations are commonly treated with (EGFR)-tyrosine kinase inhibitors (EGFR-TKIs). An experimental study demonstrated reduced expression of ABCC5 in EGFR-TKI-resistant cells, which may be associated with increased sensitivity to cytotoxic drugs such as gemcitabine and vincristine in EGFR-TKI-resistant cells (Hamamoto et al., 2017). Another investigation on the connection between gemcitabine sensitivity and ABCC5 expression levels in NSCLC cell lines revealed that gemcitabine sensitivity was mediated by the ABCC5 inhibitor zaprinast. Reducing ABCC5 expression also altered the cytotoxicity of gemcitabine, suggesting that NSCLC gemcitabine sensitivity is determined by ABCC5 expression and is linked to gemcitabine efflux (Oguri et al., 2006). Furthermore, researchers found that Adriamycin-resistant cell lines exhibit overexpression of ABCC5 and SMRP in lung cancer as well as Adriamycin-induced expression of these genes (Yoshida et al., 2001).

### Liver cancer

Melatonin is an indoleamine neurohormone. It is considered to be a safe antitumour drug (Nabih et al., 2023). One study revealed that the IC_50_ of melatonin (13.4 mM) reduces the expression of the ABCC5 gene, which mediates drug resistance in Adriamycin-resistant HepG2 cells (Hamed et al., 2023). A multitarget TKI known as sorafenib is the current treatment of choice for hepatocellular cancer. According to a previous study, sorafenib-resistant HCC cells exhibit increased ABCC5 expression, which is associated with a poor clinical prognosis (Huang et al., 2021). According to our findings, reducing ABCC5 expression greatly decreased HCC cell resistance to sorafenib (Huang et al., 2021). Additional research demonstrated that sorafenib-induced ABCC5 expression requires activation of the PI3K/AKT/NRF2 signalling pathway (Huang et al., 2021). By stabilizing the SLC7A11 protein, increasing glutathione (GSH) within the cell, and preventing the accumulation of lipid peroxides, ABCC5 prevents ferroptosis (Huang et al., 2021). Finally, it has been shown that sorafenib exhibits greater anticancer activity when ABCC5 is inhibited (Huang et al., 2021). Sorafenib resistance may be overcome clinically based on the findings of this study.

Let-7c was found to inhibit ABCC5 expression. Let-7c is a bioengineered miR agent that is useful for the treatment of HCC (Jilek et al., 2019). Several researchers have explored the effects of combining let-7c-5p and 5-FU on hepatocellular carcinoma cells using cellular experiments and analytical methods and investigated the underlying mechanisms. According to results, let-7c-5p decreases ABCC5 protein expression, thereby amplifying the cytotoxic effects of 5-FU and increasing its intracellular accumulation (Jilek et al., 2020). These findings provide a theoretical basis for combination therapy in hepatocellular carcinoma and reveal a potential mechanism for the synergistic effect of let-7c-5p and 5-FU.

### Colorectal cancer

Treatment with 5-FU promotes chemoresistance by upregulating ATP-binding cassette subfamily B member 1 (ABCB1) and ABCC5, which contributes to drug efflux and leads to acquired resistance in colon cancer (Wang et al., 2015). Some researchers have assessed the effect of different treatments on CSCs by establishing a colon cancer model. The animals were divided into different treatment groups, and the percentage of CSCs was measured by isolating colon cells (Rani et al., 2021). Subsequently, 5-FU and 5-FdUMP concentrations were assayed to evaluate the impact of various therapies on drug concentrations (Rani et al., 2021). In conclusion, the impact of various treatments on ABCB1 and ABCC5 transporter expression levels was evaluated using a variety of techniques, including immunohistochemistry and immunoblotting (Rani et al., 2021). The findings revealed that ABCB1 and ABCC5 expression was mildly increased by 1,2-dimethylhydrazine (DMH)/dextran sulfate sodium (DSS) treatment but was significantly increased by 5-FU treatment alone (Rani et al., 2021). However, in animals cotreated with 5-FU and fish oil, ABCB1 and ABCC5 expression levels were reduced (Rani et al., 2021).

Combination of PI3K inhibitor and 5-FU significantly inhibits ABCC5 expression in colorectal cancer cells. The PI3K/AKT/mTOR signalling pathway regulates a variety of cellular activities, including cell proliferation, cell cycle progression, survival, and apoptosis, and many colorectal cancers overactivate this signalling pathway (El-Daly et al., 2023). El-Daly et al. evaluated the effect of PI3K inhibitors on 5-FU cytotoxicity. The results showed that when these drugs were used alone, they had a weaker inhibitory effect even at high doses (El-Daly et al., 2023). Based on these results, the researchers chose two doses that had lower cytotoxicity (El-Daly et al., 2023). Next, the cells were exposed to different doses of 5-FU (3.12–100 μM) combined with low doses of LY294002 (25 μM) or PI-103 (2.5 μM). LY294002 is a PI3K inhibitor that blocks the PI3K/AKT/mTOR signalling pathway, enhancing cancer cell sensitivity to chemotherapy and promoting apoptosis. The simultaneous treatment with 5-FU (25 μM) + LY294002 (25 μM) yielded the best effects (El-Daly et al., 2023). Further experimental results showed that this combination regimen significantly reduced p-AKT and p-mTOR expression as well as ABCG2 and ABCC5 expression, which led to increased cellular uptake and 5-FU concentrations (El-Daly et al., 2023). This finding provides evidence for enhancing chemosensitivity by targeting survival signalling pathways.

MiR-361 enhances chemosensitivity of colon cancer cells by regulating ABCC5. MiR-361 expression was decreased in 5-FU-resistant HCT116 and HT29 colorectal carcinoma cells (Zhang et al., 2019). Zhang et al. reported that resistant cells were more susceptible to 5-FU when miR-361 was overexpressed (Zhang et al., 2019). Further experiments indicated that miR-361 overexpression inhibited drug-resistant cell colony formation and increased apoptosis (Zhang et al., 2019). Results of the experiment revealed that the target gene of miR-361 was Forkhead Box M1 (FOXM1). Furthermore, when FOXM1 was knocked down, 5-FU was more effective at killing drug-resistant colon cancer cells (Zhang et al., 2019). Additionally, ABCC5 and ABCC10 were shown to be downstream effector genes of miR-361 (Zhang et al., 2019). Taken together, these findings indicate that miR-361 regulates the FOXM1-ABCC5/10 pathway to enhance chemosensitivity (Zhang et al., 2019).

### Pancreatic carcinoma

One factor contributing to the high mortality of pancreatic cancer (PC) patients is resistance to first-line chemotherapeutic drugs. One of the main clinical issues with pancreatic ductal adenocarcinoma (PDAC) is gemcitabine resistance (Amponsah et al., 2017). MiR-210 inhibits ABCC5 expression and synthesis by binding to its 3′ UTR (Amponsah et al., 2017). Observations in three PDAC cell lines as well as in human PDAC and normal pancreatic tissues confirmed that high ABCC5 expression was associated with downregulated miR-210 expression and correlated with gemcitabine resistance (Amponsah et al., 2017). According to this study, pancreatic tumour malignancy and gemcitabine resistance are correlated with miR-210 downregulation and ABCC5 overexpression, which can be inhibited by increasing miR-210 levels (Amponsah et al., 2017). In another experiment, a new nitric oxide-releasing gemcitabine precursor (NO-GEM) was synthesized by partially adding an NO donor to gemcitabine using an appropriate linker (Masetto et al., 2020). In chemotherapy-resistant PDAC cells, NO-mediated nitration inhibits ABCC5 activity and enhances the cytotoxicity of the 5b precursor drug (a type of NO-GEM) (Masetto et al., 2020).

5-FU chemotherapy is currently used for the treatment of PC, but most patients eventually develop chemoresistance. PC is associated with obesity, which influences 5-FU effectiveness through the release of the adipokine leptin (Stolzenberg-Solomon et al., 2015). Leptin increases ABCC5 and ABCC11 expression levels in 5-FU-treated PC tumour spheres, and this effect is dependent on Notch signalling and contributes to the leptin-mediated inhibition of 5-Fu cytotoxicity in PC tumour spheres (Harbuzariu and Gonzalez-Perez, 2018).

### Ovarian cancer

Inhibition of ABCC5-mediated drug resistance by MiR-128 was also observed in ovarian cancer. Platinum-based chemotherapy is the standard treatment for advanced ovarian cancer. Studies have shown that ovarian cancer tends to be resistant to cisplatin treatment. Decreased miR-128 expression in ovarian cancer is closely related to cisplatin resistance (Li et al., 2014). Researchers have shown that SKOV3/CP cells exhibit reduced miR-128 expression levels compared with the parental SKOV3 cell line, which is cisplatin resistant (Li et al., 2014). Researchers subsequently observed changes in miR-128 expression in SKOV3, OVCAR3, and PEO14 cells after cisplatin treatment and found that miR-128 expression decreased in a cisplatin concentration-dependent manner in these cell lines (Li et al., 2014). Subsequently, scientists overexpressed miR-128 to resensitize SKOV3/CP cells to cisplatin, and the expression levels of proteins linked to cisplatin resistance, including ABCC5 and Bmi-1 were reduced (Li et al., 2014). Moreover, an miR-128 inhibitor enhanced cisplatin resistance in SKOV3 cells (Li et al., 2014). Furthermore, SKOV3/CP tumours were inhibited more effectively by cisplatin combined with miR-128 than by cisplatin alone (Li et al., 2014). In treated tumour tissues, Bmi-1 and ABCC5 expression was reduced as the cisplatin concentration increased (Li et al., 2014). According to previous studies, miR-128 could represent a promising therapeutic target.

### Prostate cancer

ABCC5 expression is significantly elevated in primary PCa, metastatic PCa, and desmoplasia-resistant PCa. Additionally, ABCC5 overexpression is associated with poor biochemical recurrence and overall survival in PCa patients according to a study on the regulation of PCa progression by ABCC5 (Zhang et al., 2018). The experimental results indicated that ABCC5 inhibition suppressed PCa cell proliferation, migration, and invasion (Zhang et al., 2018). This study revealed that miR-516a-3p could directly target the ABCC5 gene and that its expression was considerably downregulated in PCa (Zhang et al., 2018). The overexpression of miR-516a-3p mimicked the phenotypic changes induced by ABCC5 inhibition, and ABCC5 overexpression significantly reversed these inhibitory effects (Zhang et al., 2018). It was also found that miR-516a-3p controls cancer cell sensitivity to Adriamycin and doxorubicin by targeting ABCC5 (Zhang et al., 2018). Another study explored the downstream mechanism of ABCC5 in regulating tumorigenesis and development. ABCC5 promotes tumorigenesis by binding and inhibiting the protein degradation of its downstream molecule CDK1, thereby promoting the phosphorylation of AR at Ser81 by CDK1 and activating the transcriptional activity of AR for target genes (Ji et al., 2021). Furthermore, the efficacy of enzalutamide on prostate cancer cells was markedly improved by treatment with a CDK1 inhibitor or CDK1 knockdown (Ji et al., 2021).

According to another report, increased ABCC5 expression in CRPC is related to enzalutamide resistance; nevertheless, ABCC5-mediated enzalutamide resistance is enhanced by upregulating P65/AR-V7 expression, not by its traditional drug efflux function (Chen et al., 2022). This finding implies that ABCC5, which might reduce enzalutamide resistance by preventing P65/AR-V7 overexpression, may be a potential therapeutic target in CRPC.

### Cervical cancer

Most cervical cancer patients exhibit adequate sensitivity to chemotherapeutic drugs such as paclitaxel during the first phase of treatment. However, acquired resistance may occur after short-term treatment. Previous studies have found significant overexpression of FOXM1 and ABCC5 in cervical cancer cells (Hou et al., 2022). Both of these genes are associated with drug resistance in cervical cancer cells (Hou et al., 2022). Subsequently, researchers confirmed that FOXM1 is bound to the ABCC5 gene promoter (Hou et al., 2022). In paclitaxel-resistant Caski/Taxol cells, FOXM1 showed a stronger affinity for the ABCC5 gene promoter (Hou et al., 2022). Direct regulation of ABCC5 transcription potentially contributes to FOXM1-mediated drug resistance (Hou et al., 2022). Using luciferase reporter gene experiments, the regulatory effect of FOXM1 on ABCC5 gene transcription was further confirmed (Hou et al., 2022). FOXM1 upregulation enhanced the luciferase activity of the ABCC5 gene promoter, while FOXM1 downregulation decreased it (Hou et al., 2022). This finding further confirmed the regulatory effect of FOXM1 on ABCC5 gene transcription. This study revealed that FOXM1 regulates ABCC5 transcription, improving drug resistance in cervical cancer cells (Hou et al., 2022). This study provides new insight into the mechanisms of drug resistance in cervical cancer cells.

### Nasopharyngeal carcinoma

Nasopharyngeal carcinoma is treated with paclitaxel as the first-line chemotherapeutic regimen (Joerger, 2016). However, acquired resistance leads to treatment failure. The development of paclitaxel resistance is mediated by the efflux of drugs by ABC transport proteins and survival signalling activated by forkhead box molecules (Zhao et al., 2014). One study showed that nasopharyngeal cancer cell resistance to paclitaxel is significantly regulated by the FOXM1-ABCC5 axis (Hou et al., 2017). In one study, researchers found that paclitaxel resistance in nasopharyngeal carcinoma cells is influenced by FOXM1 and ABCC5 (Hou et al., 2017). Moreover, FOXM1 overexpression and ABCC5 upregulation are correlated with cell resistance to paclitaxel (Hou et al., 2017). Additionally, blocking FOXM1 and ABCC5 gene expression may considerably improve the susceptibility of cells to paclitaxel (Hou et al., 2017). A thorough analysis of the role and regulatory mechanism of the FOXM1-ABCC5 axis is presented in this study, which addresses the resistance of nasopharyngeal carcinoma cells to paclitaxel and provides a foundation for future investigations and the treatment of nasopharyngeal carcinoma.

## ABCC5 and prognosis of cancer

ABCC5 is associated with the survival and prognosis of some cancers. By performing survival analysis on clinical data of 23 types of cancer patients in the database, researchers found that the expression of ABCC5 affected the survival rate of testicular cancer patients aged 30–40 years (Kadioglu et al., 2020). Moreover, the survival rate of breast cancer patients with high ABCC5 expression over 50 years old was lower than that of patients under 50 years old (Kadioglu et al., 2020).

A study on HCC analysis RNA-Seq data from the TCGA database and matched prognostic/clinicopathologic data. The results showed that HCC patients with high ABCC5 expression tended to have a shorter median survival time (Zhou et al., 2022). Another study also demonstrated that the expression of ABCC5 was different in different pathological stages and different T stages of HCC (Chen et al., 2021a). ABCC5 was significantly differentially expressed in HCC stage 1 and stage 3 (Chen et al., 2021a). The expression level of ABCC5 was higher in stage 3 than in stage 1 (Chen et al., 2021a). Meanwhile, the expression of ABCC5 was different between T1 and T3, and the expression level of T3 was also higher than that of T1 (Chen et al., 2021a). This may indicate that elevated ABCC5 expression levels are closely associated with HCC progression. This study also suggests that ABCC5 may be an independent prognostic factor for HCC (Chen et al., 2021a).

Bioinformatics analysis showed that the overexpression of ABCC5 in ovarian malignant tumours was significantly higher than that in ovarian surface epithelium (Ting et al., 2022). And ABCC5 was significantly higher in patients with stage III-IV ovarian cancer than in patients with stage I-II (Ting et al., 2022). High ABCC5 expression is associated with poor overall survival (OS) in ovarian cancer patients (Ting et al., 2022). Kaplan-Meier analysis showed that the OS of patients with negative ABCC5 expression was significantly higher than that of patients with positive ABCC5 expression (Ting et al., 2022).

A retrospective study showed that the immunohistochemical expression of ABCC5 may have prognostic significance in patients with glioblastoma (GBM) (Alexiou et al., 2012). It was found that patients with an ABCC5 index of more than 11% had a significantly shorter survival compared to patients with an ABCC5 index of less than or equal to 11% (10.5 months versus 18 months, *p* = 0.0002) (Alexiou et al., 2012). In multivariate analysis, ABCC5 index and extent of tumour resection were identified as factors with independent prognostic power (Alexiou et al., 2012).

ABCC5 overexpression was significantly associated with PCa progression and poor prognosis (Zhang et al., 2018). ABCC5 was found to be significantly increased in patients with BCR (biochemical recurrence) compared to those without BCR at 3 and 5 years, respectively (Zhang et al., 2018). ABCC5 was significantly increased in higher grade, higher stage, and higher Gleason score patients relative to lower grade, lower stage, and lower Gleason score patients (Zhang et al., 2018). Further studies showed that ABCC5 protein overexpression was significantly associated with lymph node metastasis, clinical stage, preoperative PSA, Gleason score and BCR (Zhang et al., 2018). Patients with high ABCC5-expressing PCa had shorter overall or BCR-free survival compared with patients with low ABCC5 expression (Zhang et al., 2018). ABCC5 is an independent prognostic factor for BCR-free survival and overall survival (Zhang et al., 2018).

## Discussion

**FIGURE 3:**
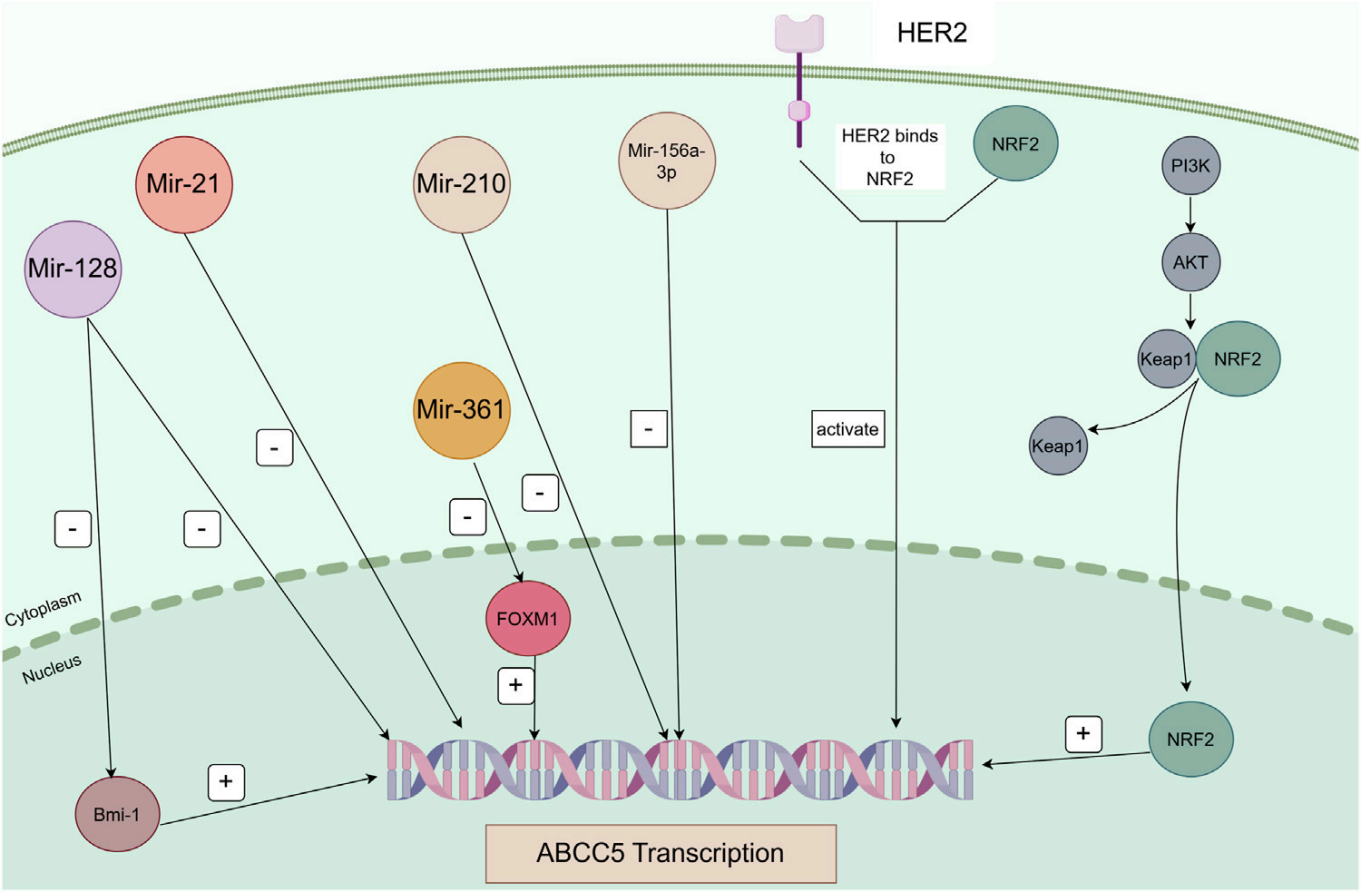
Regulatory pathways of ABCC5 in cancer. Mir-128 directly represses Bmi-1 and ABCC5 transcription, and Bmi-1 directly regulates ABCC5 transcription. Mir-21, Mir-210, and Mir-516a-3p directly repress ABCC5 transcription. Mir-361 negatively regulates FOXM1, and FOXM1 positively regulates the transcription of its target gene ABCC5. ABCC5 is a transcriptional target of NRF2. Sorafenib induces ABCC5 expression through activation of the PI3K/AKT/NRF2 signalling pathway. Binding of HER2 and NRF2 activates transcription of ABCC5.

Cancer therapy has become increasingly challenging due to MDR, and the main mechanism leading to MDR is the efflux of chemotherapeutic agents through the overexpression of drug efflux pumps on cancer cells. Studies on transporter proteins in recent years have focused on P-gp, MRP1, and BCRP. However, in this review, we focus on ABCC5. Numerous studies have demonstrated that ABCC5 exhibits increased expression in a variety of cancers. And ABCC5 is associated with the survival and prognosis of some cancers. In addition, high ABCC5 expression is closely associated with drug resistance in cancer therapy. ABCC5 is regulated by corresponding regulatory pathways in a variety of cancers; thus, we organized information regarding relevant regulatory pathways (Table 2). And Figure 3 is plotted for a better understanding. A key role of ABCC5 is to facilitate drug efflux and reduce effective concentrations of drugs in cancer cells. However, a study revealed that ABCC5 causes prostate cancer drug resistance through nondrug efflux mechanisms. In conclusion, ABCC5 is a very promising target for cancer therapy, and drug resistance in cancer therapy may be overcome or reduced with inhibitors or other strategies against ABCC5. However, there are still many challenges to be faced in realizing this goal. Here are some of the major challenges in current research, inhibitors that have been developed, and possible solutions.

**TABLE 2:** Regulatory pathways of ABCC5 in different cancer.

Type of cancer	The role of ABCC5	Related pathways/mechanisms	Impact on drug resistance	Citation
Breast cancer	Drug efflux	SLC34A2-Bmi1-ABCC5 signalling pathway, miR-128-bmi1-ABCC5 pathway	Upregulation of pathways reduces the effect of chemotherapeutic drugs and promotes drug resistance	Zhu et al. (2023), Ge et al. (2016)
Lung cancer	Drug efflux	BRM270 negatively regulates ABCC5 and Bmi-1 expression by upregulating miR-128	Enhancing resistance to chemotherapeutic drugs	Gu et al. (2021)
Liver cancer	Drug efflux, affects lipid peroxidation	PI3K/AKT/NRF2 signalling pathway upregulates ABCC5 expression	Reduces the intracellular concentration of chemotherapeutic drugs and promotes sorafenib resistance	Huang et al. (2021)
Colorectal cancer	Drug efflux	Upregulation of the miR-361-FOXM1-ABCC5/10 pathway	Reduces the effectiveness of chemotherapy drugs, enhances drug resistance	Zhang et al. (2019)
Pancreatic carcinoma	Drug efflux	MiR-210 negatively regulates ABCC5	Decreased miR-210 expression enhances gemcitabine resistance in pancreatic cancer	Amponsah et al. (2017)
Ovarian cancer	Drug efflux affects cisplatin sensitivity	MiR-128 negatively regulates ABCC5 and Bmi-1 expression	Downregulation of the pathway reduces the effect of cisplatin treatment and promotes drug resistance	Li et al. (2014)
Prostate cancer	Drug efflux, affects the treatment response	Inhibition of the miR-516a-3p pathway negatively regulates ABCC5 and ABCC5-CDK1-AR regulatory pathways	It is related to poor treatment response and drug resistance	Zhang et al. (2018), Ji et al. (2021)
Cervical cancer	Drug efflux	Upregulation of the FOXM1-ABCC5 axis	Enhanced resistance to drugs such as paclitaxel	Hou et al. (2022)
Nasopharyngeal carcinoma	Drug efflux	Upregulation of the FOXM1-ABCC5 axis	Promotes resistance to paclitaxel	Hou et al. (2017)

Although ABCC5 is a promising therapeutic target, the development of specific inhibitors against it is still in the early stages to date. A number of compounds have been identified that inhibit ABCC5 activity in a variety of experimental models, but no ABCC5 inhibitors have been approved for clinical use. Table 3 lists some of the ABCC5 inhibitors and their associated mechanisms in current studies.

**TABLE 3:** ABCC5 inhibitor.

Inhibitor	Mechanisms of action	Clinical research stage	Applications and limitations	Ref
MK-571	Broad-spectrum MRP family inhibitor that prevents ABCC5 from translocating chemotherapeutic drugs	Experimental research	Mainly used in in vitro models, lacks specificity, inhibits a wide range of MRPs, may lead to nonspecific side effects	Bertollotto et al. (2018), Yang et al. (2010)
Probenecid	Inhibition of organic anion transporters, including ABCC5	Experimental research	Used to study drug metabolism and excretion in cancer, clinical use is mainly for gout treatment and lacks specificity	Yang et al. (2010)
Indomethacin	Non-steroidal anti-inflammatory drug showing inhibition of ABCC5	Experimental research	Clinical application as an anti-inflammatory drug, not well-studied as an ABCC5 inhibitor	Adema et al. (2014)
Sildenafil	Inhibits cGMP efflux by binding to ABCC5	Clinical research	Enhancing the efficacy of anticancer drugs. Although sildenafil has some inhibitory effect on ABCC5, its main pharmacological effect is to inhibit PDE5	Haider et al. (2021), Sager et al. (2012)
Curcumin	Inhibition of several ABC transporters including ABCC5	Clinical research	Curcumin has low water solubility and poor *in vivo* stability, which limits its bioavailability and subsequent anticancer effects	Xu et al. (2021), Li et al. (2011)

The development of specific inhibitors against ABCC5 faces the following major challenges. First, the problem of specificity: most of the currently available inhibitors are broad-spectrum MRP family inhibitors that lack high specificity for ABCC5. For example, MK-571 inhibits not only ABCC5, but also ABCC1 and ABCC4, which can lead to untargeted side effects. Second, side effects: since ABCC5 is not only expressed in cancer cells but also functions in normal tissues, one of the main challenges in developing specific inhibitors is how to minimize the effects on normal cells. Broad-spectrum inhibitors such as Probenecid also act in normal cells and may cause unwanted toxicity and side effects. Third, drug tolerance and drug-drug interactions: Cancer cells may compensate for the inhibitory effects of ABCC5 through other resistance mechanisms, and the developed inhibitors may face rapidly emerging drug tolerance.

Although ABCC5 inhibitors have shown reversal of chemoresistance in laboratory models, inhibitors targeting ABCC5 have not yet entered large-scale clinical trials. The following are areas to focus on in the future. Preclinical models: Most of the current studies are limited to *in vitro* cellular experiments and animal models, and a wider range of preclinical models are needed to validate the effect of inhibiting ABCC5 on different cancer types. Individualized therapy: As the expression level of ABCC5 varies in different tumours and patients, conducting individualized therapy research is a key direction for the future. By detecting the level of ABCC5 expression in a patient’s tumour, a treatment plan can be tailored to the patient. Combination therapy: A single therapy targeting ABCC5 may not be sufficient to completely overcome resistance. Combining ABCC5 inhibitors with other chemotherapeutic or targeted agents may be an important strategy for the future. For example, combining ABCC5 inhibitors with immunotherapy is expected to enhance overall efficacy.

The following are possible solutions and future directions. First, advances in structural biology and computer-aided drug design have made it possible to develop specific inhibitors of ABCC5. In-depth study of the crystal structure of ABCC5 could help to design inhibitors with higher selectivity and lower toxicity. Second, using high-throughput drug screening technology, more potential ABCC5 inhibitors can be screened and validated on a large scale. Third, the efficacy of ABCC5 inhibitors in combination with existing chemotherapy regimens should be explored in early preclinical and clinical stages. Its safety and efficacy in a variety of tumours also remain to be verified. Finally, individualized treatment plans should be formulated according to the expression of ABCC5 in patients' tumours, combined with gene detection and targeted therapy to optimize the therapeutic effect.
